# Agricultural Pollution as a Driver for the Ecological Status of Rivers in the Long‐Term Perspective of Nida River Assessment (Central Europe)

**DOI:** 10.1002/ece3.71541

**Published:** 2025-06-16

**Authors:** A. Cieplok, R. Czerniawski, A. Spyra

**Affiliations:** ^1^ Faculty of Natural Sciences, Institute of Biology, Biotechnology and Environmental Protection University of Silesia Katowice Poland; ^2^ Department of Hydrobiology, Institute of Biology University of Szczecin Szczecin Poland

**Keywords:** agricultural land use, rivers, water management, water policy, water quality, WFD

## Abstract

Benthic invertebrates utilized as indicators of river ecological status were examined within the river situated in agricultural catchments as a model system to assess the significance of agricultural pollution in accordance with the European Water Framework Directive (WFD). The extensive study revealed variations in the physicochemical parameters of the water over the years, including pH, NO_3_, NH_4_, total dissolved solids, salinity, chlorides, and dissolved oxygen. Notably, the maximum density of invertebrates consistently showed a significant increase during the spring compared to the autumn, while multimetric index values remained comparable. The ecological status fluctuated over the years, with classifications ranging from good and moderate in 2014, moderate in 2017, to good and poor in 2020 and 2023. The significance of the long‐term monitoring study lies in the evolving impact of parameters on the river's ecological status, index component metrics, and MMI values. Additionally, the number of variables exhibiting a significant relationship with individual component metrics has expanded over time. Seasonal variations, as indicated by redundancy analysis results, demonstrated that chlorophyll *a* and chlorides (in spring) and conductivity (in the autumn) significantly influenced seven metrics and the water quality class. While differences in component metric values were observed in specific years, the water quality class remained comparable over the long term in the two seasons. In the seasons, the MMI_PL value and the quality class were comparable. However, individual component metrics were influenced by various environmental factors, highlighting the importance of considering these factors in interpreting the results of benthic macroinvertebrate studies.

## Graphical abstract

Benthic invertebrates utilized as indicators of river ecological status were examined within the river situated in agricultural catchments as a model system to assess the significance of agricultural pollution in accordance with the European Water Framework Directive (WDF). While differences in component metric values were observed in specific years, the water quality class remained comparable over the long term in the two seasons. In the seasons, the MMI_PL value and the quality class were comparable. However, individual component metrics were influenced by various environmental factors, highlighting the importance of considering these factors in interpreting the results of benthic macroinvertebrate studies.

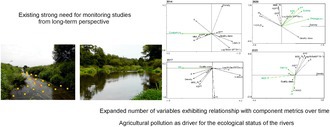

## 1 Introduction

The long‐term analysis of the utilization of ecological indicators is infrequently explored in the context of employing benthic invertebrates for evaluating the ecological status of the rivers. Rivers, regarded as valuable ecosystems with high ecological significance (Nguyen et al. 2018), undergo consistent natural changes. Yearly floods and periods of low flow alter the morphological conditions of the riverbed, the vegetation along its banks, and the communities of aquatic organisms within the river valley (Prus et al. 2018). In addition to these natural influences, land usage, human activities, and anthropogenic pressure exert a significant impact on river ecosystems (Krodkiewska et al. 2022; Ribeiro et al. 2022). From an ecological standpoint, human activities have extensive and diverse effects on the functioning of water systems (Fowler et al. 2013; Best and Darby 2020).

Deteriorating water quality in urbanized regions is linked to a substantial presence of hardened and impermeable surfaces. If these surfaces surpass 5% to 10% of the entire catchment area, they can significantly degrade the ecological condition of the stream (Schiff and Benoit 2007). The migration of people to cities is an additional factor contributing to increased water pollution, driven by elevated water consumption (Mouri et al. 2011), leading to a higher production of domestic sewage (Halecki and Stachura 2020). Human activities associated with industrial plants release various pollutants into the atmosphere (Manisalidis et al. 2020), directly and indirectly impacting the quality of surface and groundwater. These activities, combined with agricultural practices, contribute to eutrophication. Urbanization and nutrient pollution serve as crucial indicators of ecological degradation. The collecting impact of human actions negatively affecting river conditions exerts multiple pressures on aquatic systems, undermining their biodiversity and ecological functioning (Grizzetti et al. 2017). Agricultural pollution of rivers poses an escalating challenge, contributing to the progressive deterioration of river ecosystems (Evans et al. 2019). Approximately 80% of agricultural pollutants find their way into rivers, originating from sources such as pesticides and artificial fertilizers, primarily nitrogen and phosphorus compounds (Mateo‐Sagasta et al. 2017; Akhtar et al. 2021). These pollutants exert adverse effects on aquatic systems, leading to eutrophication, salinization, alterations in water quality, and ultimately resulting in the decline and migration of native species, alongside the proliferation of invasive species.

Only approximately 40% of European rivers exhibit good ecological conditions (Grizzetti et al. 2017; Kristensen et al. 2018). In Poland, a mere 10% of rivers attain good or very good ecological status, while 60% hold a moderate status, and 30% are classified as having a poor or bad ecological status in the period from 2014 to 2019 (Data from the Chief Inspectorate of Environmental Protection in Poland; GIOŚ 2020). The European Union has embraced an ambitious water policy aimed at alleviating pressures and achieving a good ecological status for rivers. The original objective that was assumed was to achieve at least good ecological status of water in water bodies by 2015 and currently by 2027, in the event of nonfulfillment. The assessment of the ecological status of flowing waters holds paramount importance due to the enforcement of laws by countries within the European Union. Legal regulations like the Nitrates Directive focused on safeguarding waters against pollution caused by nitrates of agricultural origin and the Water Framework Directive (WFD) are integral to this initiative. The WFD, serving as a document outlining the principles of community action in water policy, aims to establish a framework for the qualitative and quantitative protection of marine (transitional and coastal) waters, underground waters, and inland surface waters (Benson et al. 2014; Halecki and Stachura 2020). The documentation is reviewed and updated in a 6‐year planning cycle and forms the basis for making decisions affecting the status of water resources and the principles of their management in the future. The delineation between good and moderate ecological status is particularly significant in achieving the environmental goals set by the WFD. Consequently, the WFD necessitates operational measures to enhance water quality in areas at immediate risk of falling short of environmental requirements (Bis and Mikulec 2013).

The assessment of the ecological status or ecological potential of surface waters, as per the Water Framework Directive (WFD), involves the examination of biological indicators, which constitute the fundamental element supported by hydromorphological and physicochemical elements. Among these components, phytobentos, fish, and macrophytes are included, as well as the composition and abundance of benthic macroinvertebrates, which are sensitive to changes in the aquatic environment induced by anthropogenic pressures. The reaction of benthic macroinvertebrates to environmental stressors plays a pivotal role in the ecological monitoring of rivers. The Polish Multimetric Index MMI_PL serves as the standard methodology for classifying the ecological state of waters, developed based on the existing Multimetric Intercalibration Index ICMi (Bis et al. 2020). Analyzing the response of benthic macroinvertebrate communities in comparison to reference conditions enables the estimation of the ecological status of rivers and the categorization of water quality.

Benthic macroinvertebrates, widely acknowledged as excellent indicators for the biological assessment of flowing water quality (Bis et al. 2020; Kaboré et al. 2022; Mao et al. 2023), serve as valuable tools in evaluating water quality and overall aquatic ecosystem health (Tampo et al. 2021). These institutions conduct monitoring studies only at selected points, resulting in a lack of comprehensive data along the entire length of rivers, especially from a long‐term perspective. Providing reliable information and data on the ecological status (or ecological potential) and chemical status of rivers is crucial for water management in river basins, including protection against eutrophication and anthropogenic pollution. Assessing the various pressures on aquatic systems and their impact on ecological status is challenging, particularly on a large scale, yet essential for planning effective policies (Grizzetti et al. 2017; Pistocchi et al. 2017; Begum et al. 2023; Van de Pol et al. 2023).

This study had three primary objectives: (1) illustrate shifts in the ecological status of a lowland Nida river—left tributary of the Vistula, serving as a model with agricultural land use, localized in Southern Poland; (2) evaluate the use of benthic invertebrates as an ecological indicator over the long term; and (3) assess the extent to which the EU water policy target of achieving a good ecological status has been realized in the examined river. Our study contributes to knowledge of riverine ecosystem functioning because it reflects the river's ecological health based on ecological conditions and invertebrates. River Nida served as a model environment to assess the long‐term changes in water quality in agricultural areas, especially in the context of regionalization, which is a helpful tool for the rational management of water resources (Wałęga et al. 2022). This study provides the first summary of benthic invertebrate‐based metrics in a long‐term context, in the entire Nida river course in Poland, not only from a single sampling point. Our study uniquely assesses the macroinvertebrate community dynamics in the Nida River, revealing significant changes over the years that highlight the river's ecological functioning. It offers a comprehensive, updated evaluation of the benthos community structure, providing critical insights into the impacts of land use alterations and human activities on freshwater ecosystems. The bio‐monitoring of rivers using aquatic organisms is well‐established in most developed countries (Carter et al. 2017), and our results constitute a comparative basis for changes in the ecological state of the river in agricultural catchment.

## 2 Materials and Methods

### 2.1 Study Area of the Model River With an Agricultural Catchment

**FIGURE 1:**
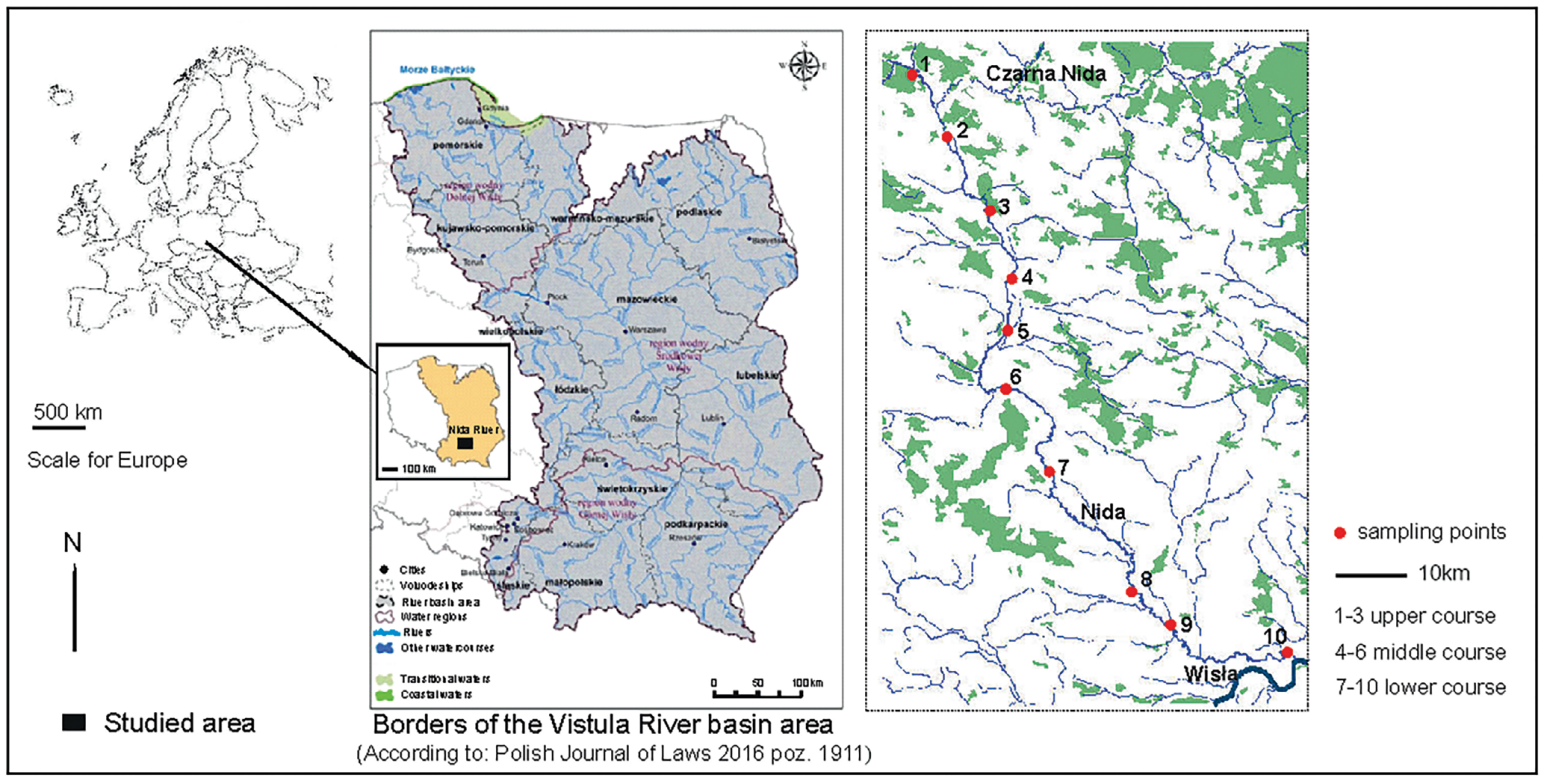
Locality of the studied area in Central Europe (Southern Poland).

The Nida River (National Code RW20001121699, RzN type) was chosen for a monitoring study as a representative river with agricultural land use, emphasizing a long‐term perspective in using benthic invertebrates to evaluate ecological status (Table 1S). This river, on the left bank of the Vistula River, spans 151.2 km in length with a catchment area of 3865.4 km^2^. Situated in southern Poland, it traverses three macroregions: the Małopolska Upland, the Przedborska Upland (Biała Nida River catchment area), the Kielecka Upland (Czarna Nida River catchment area), and the Niecka Nidziańska (Nida River catchment area) (Kondracki 2000; Strużyński 2006) (Figure 1). The Nida is a lowland river with a moderate slope, largely maintaining its natural character along a significant stretch. However, human activities have significantly altered some areas. Certain sections have undergone full riverbed regulation, while the stretch between Motkowice and Pińczów has experienced partial regulation due to drainage initiatives and meander shortening (Table 1S).

To enhance agricultural production in the Nida Valley, the Nida River underwent a transformation involving the technical regulation of its riverbed. Unfortunately, this alteration had adverse effects on flow conditions, disrupting the biological equilibrium in both the riverbed and the surrounding valley. The substantial human intervention in the form of riverbed regulation has resulted in recurrent flooding during spring thaws and autumn. In natural segments, the Nida remains a secure river, devoid of floods, ensuring the preservation of the unique natural value of the region (Łajczak 2006; Strużyński 2006). The middle section of the Nida River is embanked, and this transformation has suffered neglect and damage. Sandy bottom sediments and spring floods contribute to an increased risk of flood embankment failures. Historically, the Nida River formed numerous meanders in this area, leading to frequent damage to embankments, particularly where oxbow lakes intersected.

The Nida catchment area primarily comprises agricultural zones, with 44.26% being arable land situated beyond the reach of irrigation facilities, 16.23% covered by coniferous forests, 11.85% consisting of meadows and pastures, and 7.35% hosting sparse urban development (CLC 2018) (Table 1S). The river's catchment area features numerous watercourses forming a well‐defined hydrographic network. Owing to shifts in land use, including the discontinuation of intensive agriculture in the Nida Valley, there is an increasing consideration for restoring the natural character of this watercourse through rehabilitation efforts in the drained river valleys. These restoration works encompass extending the river course, eliminating or reconstructing a substantial portion of flood embankments, and reinstating the Nida oxbow lakes. Such endeavors aim to mitigate the risk of flooding and contribute to the preservation of the biodiversity of the river valley (Bartnik et al. 2004; Bik et al. 2006).

### 2.2 Field and Laboratory Study According to the Requirements of Monitoring Assessment (WFD 2000/60/WE)

The assessment of the river's ecological status was conducted during the spring and autumn periods from 2014 to 2023, using benthic macroinvertebrates recognized as highly recommended indicators for evaluating the quality of lotic environments. The ecological status was evaluated following the methodology for collecting multihabitat samples of benthic macroinvertebrates (RIVECOmacro) in small and medium‐sized rivers designed for ecological monitoring purposes. This method was stipulated by the WFD (2000/60/WE) (WFD 2000) and complies with the requirements for environmental quality control in European Union countries (Bis and Mikulec 2013; AQEM consortium 2002). Currently, this methodology serves as the national water quality standard and has been accepted by the Polish Committee for Standardization (PN‐EN 16150:2012E). The selection of sampling sites was based on a prior assessment of hydrological, hydrogeological, and hydrochemical materials and maps covering the length of the river. Samples were collected at each site over a distance of 100 m. While conducting fieldwork, physical and chemical water parameters were analyzed using the Hydrolab DS 5 multiprobe from the USA.

Before sampling bottom fauna assemblages, an evaluation of the heterogeneity of the bottom substrate was conducted, considering the percentage distribution of various types of mineral and organic substrate in the riverbed's bottom cover. In line with the methodology, habitats constituting less than 5% of the bottom cover at the sampling site were disregarded during sample collection. A total of 20 partial samples, covering a combined area of 1.25 m^2^, were extracted from the primary river habitats at each chosen site. Partial samples were obtained using a hydrobiological net positioned frontally in the direction of water flow. The sampling strategy aimed to establish a long‐term perspective in utilizing invertebrates as indicators of the ecological status of the model river. The collected biological material was stored in tightly sealed and labeled containers and preserved using 96% ethanol. Benthic macroinvertebrate samples collected in the field underwent a rinsing process with water through sieves with a mesh size of 0.40 mm, while larger organic matter fragments were removed from the sample. The material was evenly distributed over the surface of the cuvette‐sieve, divided into 30 fields measuring 6 cm × 6 cm. Five subsamples were randomly selected from the material, and the number of individuals was verified to achieve a count of 350 individuals. If the number of organisms obtained from the five subsamples at each site was less than 350, additional subsamples were selected, and macroinvertebrates were identified following the methodology (Bis and Mikulec 2013; AQEM consortium 2002).

Along with each sampling of invertebrates, also physicochemical water parameters, including temperature, conductivity, total dissolved solids (TDS), pH, chlorides (Cl), salinity and chlorophyll a, and dissolved oxygen (DO) concentration, nitrates (NO3), and ammonia (NH3), were measured using a HydroLAB DS probe. All parameters were measured in the field. Measuring the physicochemical parameters of the water was carried out at each sampling point along the entire river. The values of the MMI_PL index for the Nida River were determined based on the Multimetric Intercalibration Index ICMi. Each ICMi value was equated to MMI_PL = 1; otherwise, MMI_PL = ICMi, following the procedures established by the Central‐Baltic Geographical Intercalibration Group (Bis and Mikulec 2013). The MMI_PL index value is a weighted average derived from six partial metrics: ASPT_PL (average score per taxon PL), 1‐GOLD (abundance of Gastropod, Oligochaeta, and Diptera), log10 (sel_EPTD +1) (selected families from Ephemeroptera, Plecoptera, Trichoptera, and Diptera), S (the total number of families), EPT (the number of families from Ephemeroptera, Plecoptera, Trichoptera), and the Shannon–Wiener diversity index (H′) (Table 2S).

### 2.3 Statistical Analysis

The biological and chemical data underwent normality testing using the Kolmogorov–Smirnov test. Given that these data did not conform to the assumptions of normal distribution tests, a nonparametric alternative, specifically the Mann–Whitney U test in STATISTICA version 13.1 (Dell version), was used to assess differences in component MMI metrics between spring and autumn. To ascertain the significance of differences in all component metrics across the years of the monitoring study, the nonparametric Kruskal‐Wallis analysis of variance (ANOVA) test and the subsequent multiple comparisons post hoc test (STATISTICA 13.3) were utilized.

Redundancy Analysis (RDA), serving as a direct extension of multiple regression, was implemented using CANOCO software version 4.5. RDA models the impact of the explanatory matrix and summarizes linear relationships between a set of responses and explanatory variables (Ter Braak and Šmilauer 2002). The linearity or unimodality of relationships between variables was assessed using Detrended Correspondence Analysis (DCA). DCA indicated that the data exhibited a linear response to the gradient (gradient < 3 SD). The significance of relationships between water parameters and MMI_PL metrics, as well as the axes, was evaluated using the forward selection procedure (Monte Carlo permutation test). The visualization of response and explanatory variables was accomplished using CanoDraw (CANOCO software version 4.5) based on statistically significant variables.

The Spearman rank correlation coefficients (*r*_*s*_) were used to analyze the relationships between the density of macroinvertebrates and physico‐chemical parameters of the water and MMI_PL metrics (Statistica version 13.1, Dell version). In all statistical analyses, only the significant results were taken into account (*p* < 0.05).

## 3 Results

The model river under study with the agricultural catchment exhibited variations in physico‐chemical water parameters (Table 1). The pH values of water ranged from 7.6 to 8.9, and there were significant differences between the study years (ANOVA K‐W; H(3, *N* = 40) = 22.335, *p* = 0.000). The NO_3_ values ranged from 0.2 mg/dm^3^ to 19.6 mg/dm^3^, mean 3.7–10.5 (ANOVA K‐W; H(3, *N* = 40) = 23.642, *p* = 0.0000), while NH_4_ ranged from 0.12 mg/dm^3^ to 0.8 mg/dm^3^, mean 0.2–0.47 (ANOVA K‐W; H(3, *N* = 40) = 26.847, *p* = 0.0000). Chlorophyll *a* values varied along the river course, displaying an increasing trend, with the highest value (19 μg/L) observed at sampling site 10, mean value 16.2 (Table 1). Significant differences were found in temperature (ANOVA K‐W; H(3, *N* = 40) = 15.688, *p* = 0.0013), total dissolved solids (ANOVA K‐W; H(3, *N* = 40) = 19.955, *p* = 0.0002), salinity (ANOVA K‐W; H(3, *N* = 40) =23.283, *p* = 0.000), and chlorides (ANOVA K‐W; H(3, N = 40) = 25.639, *p* = 0.000) between the study years. Dissolved oxygen ranged from 3.77 mg/dm^3^ to 7.4 mg/dm^3^, and its values differed significantly over the study period (ANOVA K‐W; H(3, *N* = 40) = 21.724, *p* = 0.0001).

**TABLE 1:** Physicochemical water properties of model river catchment area in km^2^.

	1	2	3	4	5	6	7	8	9	10
pH	7.8–8.4	7.6–8.6	7.4–8.7	7.8–8.7	7.7–8.8	7.7–8.9	7.7–8.9	7.7–8.9	7.7–8.9	7.8–8.9
	8.0÷0.26	7.9÷0.47	7.8÷0.5	8.07÷0.4	8.04÷0.5	8.05÷0.5	8.01÷0.6	8.1÷0.5	8.2÷0.5	8.2÷0.4
TDS, mg/L	205–294	198–318	219–331	235–337	230–339	240–352	238–361	221–399	250–401	231–401
	269÷43.0	254÷60.6	271÷52	283÷49	285÷53	291÷59	295÷65	300÷84	353÷69.4	356÷84.6
Temperature, °C	16.8–19.9	17.4–19.0	17.6–20.4	18.7–21.2	17.4–21.7	16.9–22.1	18.9–22.8	17.6–22.6	17.7–21.6	18.1–21.8
	18.3÷0.9	19.4÷1.4	19.03÷1.13	19.3÷1.3	18.7÷1.9	18.7÷2.4	20.4÷1.7	19.7÷2.3	19.6÷2.0	19.7÷1.9
Salinity, ‰	0.21–0.24	0.19–0.32	0.22–0.33	0.21–0.33	0.21–0.37	0.22–0.28	0.2–0.29	0.21–0.27	0.21–0.3	0.2–0.33
	0.22÷0.01	0.24÷0.05	0.26÷0.04	0.26÷0.05	0.27÷0.07	0.26÷0.02	0.25÷0.03	0.24÷0.03	0.24÷0.04	0.27÷0.05
Conductivity, μS/cm	380–459	380–497	400–517	480–527	490–530	470–549	364–532	420–564	386–603	460–648
	423÷38.8	439÷50.7	460÷47.8	494÷22.4	506÷17.0	512÷32.7	476÷77.0	499÷63.3	494÷89.4	552÷83.4
DO, mg/L	3.77–6.40	4.48–6.60	4.24–6.40	4.46–6.2	5.41–7.2	4.34–7.6	4.59–7.2	4.42–7.1	4.8–7.3	4.98–7.4
	5.04÷1.3	5.53÷0.8	5.67÷0.9	5.7÷0.8	6.6÷0.8	5.8÷1.2	6÷1.09	6.4÷1.3	6.5÷1.1	6.4÷1.1
NH_4_, mg/L	0.14–0.29	0.17–0.42	0.2–0.38	0.18–0.38	0.19–0.45	0.16–0.41	0.17–0.44	0.12–0.73	0.12–0.8	0.14–0.5
	0.2÷0.06	0.33÷0.11	0.29÷0.09	0.28÷0.1	0.3÷0.1	0.28÷0.1	0.31÷0.13	0.37÷0.27	0.47÷0.28	0.31÷0.18
NO_3_, mg/L	0.2–10.4	0.2–15.72	2.71–17.6	3.04–18.6	2.76–18.0	2.97–18.6	2.56–19.5	2.34–19	2.17–19.6	0.79–18.5
	3.7÷4.5	5.4÷6.9	7.35÷6.8	7.6÷7.3	7.0÷7.3	7.7÷7.2	8.1÷7.7	8.4÷7.4	10.5÷7.1	8.5÷8.2
Cl, mg/L	97–312	140–477	140–497	121–459	124–491	126–456	126–463	124–432	130–418	128–491
	187÷93.3	240÷160	236÷174	239÷151	244÷168	233÷150	240÷153	238÷133	247÷134	250÷163
Chlorophyll a, μg/L	4.41–6.70	3.21–3.80	3.70–3.97	4.39–5.44	5.14–6.09	7.82–8.24	9.51–10.9	10.2–16.9	11.9–15.3	14.2–19
	5.08÷1.08	3.37÷0.28	3.83÷0.13	5.07÷0.46	5.4÷0.43	7.9÷0.19	9.9÷0.66	12.1÷3.17	13.3÷1.58	16.2÷2.07

*Note:* 1–10 sampling points along the river, top row: Min–max values; lower row: Mean and standard deviation.

Abbreviations: DO, dissolved oxygen; TDS, total dissolved solids.

A positive correlation was found between the density of invertebrates and the concentration of dissolved oxygen (*r*_*s*_ = 0.3609, *p* < 0.05) and chlorophyll a (*r*_*s*_ = 0.3485, *p* < 0.05).

A total of 38,325 macroinvertebrates, spanning 74 families (including Oligochaeta), were documented in the Nida River throughout the period from 2014 to 2023. The density of benthic invertebrates varied along different sections of the river, ranging from a minimum of 280 ind./m^2^ at sampling point 1 to a maximum of 1384 ind./m^2^ at sampling point 8 (Table 2); minimal values of density were similar. The number of families also varied, ranging from 11 to 34. EPT values were lowest at site 4, while higher at site 1 and site 10. Diversity index (H′) values exhibited slight variations across sampling sites (Table 2).

**TABLE 2:** Results of the long‐term study (2014–2023) approach in the model with agricultural catchment at sampling points (1–10).

Metrics	1	2	3	4	5	6	7	8	9	10
ASPT	4.72–5.21	4.64–4.86	4.70–5.83	4.25–4.82	4.6–5.37	4.52–5.31	4.96–5.18	4.25–5.26	4.76–5.26	5.21–5.89
Log10(Sel EPTD)	0.69–2.17	0.41–1.98	0.62–1.32	0–0.76	0.53–1.38	0.69–1.99	0–1.02	0–0.86	0–1.51	0.76–1.37
1‐GOLD	0.26–0.79	0.44–0.62	0.33–0.94	0.32–0.40	0.21–0.64	0.14–0.64	0.16–0.50	0–0.69	0.16–0.53	0.21–0.38
S	18–28	24–28	12–24	17–26	11–34	21–26	22–36	17–29	15–30	18–19
EPT	3–7	3–4	3–4	1–3	2–7	1–6	3–6	1–4	1–5	5–6
H′	1.74–2.55	2.09–2.61	1.28–2.26	2.07–2.44	1.77–2.83	2.01–2.67	2.38–2.83	1.65–2.27	2.09–2.44	2.06–2.49
Density	280–1203	284–774	292–579	281–326	280–521	300–567	290–597	298–1384	284–626	304–759
MMI_PL index	0.442–0.674	0.472–0.615	0.545–0.677	0.392–0.586	0.464–0.780	0.543–0.771	0.549–0.733	0.407–0.608	0.472–0.688	0.663–0.706
Quality class	3–4	3–4	3	3–4	2–4	2–3	2–3	3–4	3–4	3
Length (km)	43	53.9	65.5	73.7	79.2	85.8	101	121.6	131.5	145.9

Abbreviations: Density, density of invertebrates; Length, length of the river in which the sampling point was selected; Quality class, ecological quality class.

**FIGURE 2:**
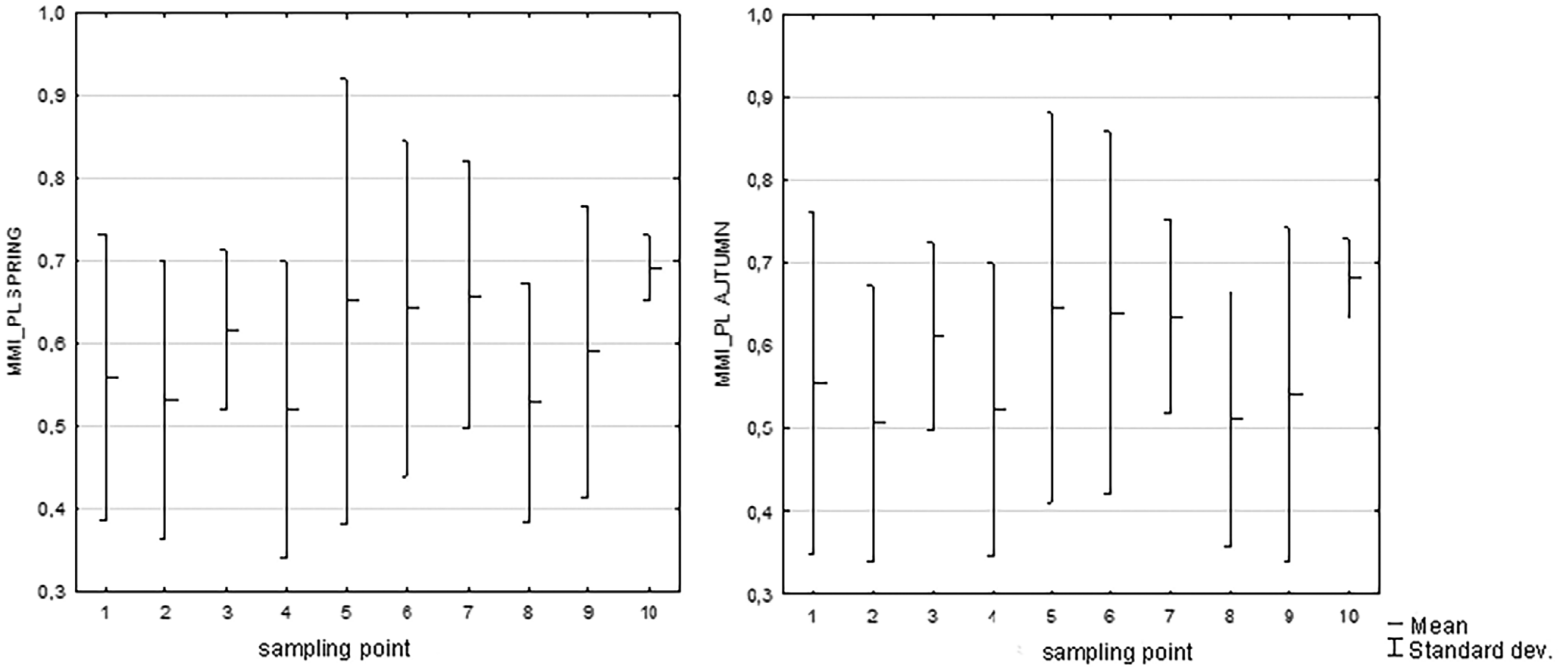
Results of the assessment of the ecological status following the MMI_PL index in two seasons in the model river.

The maximum number of individuals per m^2^ collected in spring and autumn samples varied across specific years (Table 3). In spring, the range was from 1384 in 2014 to 604 in 2020, while in autumn, it ranged from 952 in 2014 to 453 in 2023. The maximal density of invertebrates was consistently higher in the spring compared to the autumn sampling period (Table 3). The density of benthic macroinvertebrates showed statistically significant differences between spring and autumn (U Mann–Whitney test: Z = −2.22761, *p* = 0.025). However, an assessment of water quality in the Nida River based on the composition of benthic macroinvertebrates using the MMI_PL index in two seasons indicated that the index values were comparable (Table 3, Figure 2).

**TABLE 3:** Results of the long‐term study approach in the model river with agricultural catchment in the two seasons of sampling.

Year of the study	2014		2017		2020		2023	
Season of the study	I	II	I	II	I	II	I	II
ASPT	4.62–5.21	4.52–5.30	4.66–5.29	4.72–5.23	4.25–5.43	4.72–5.43	4.25–5.89	4.25–5.89
Log10(Sel_EPTD +1)	0–1.98	0–1.32	0–1.64	0–1.71	0–1.51	0–1.44	0–2.1	0–189
1‐GOLD	0.21–0.84	0.23–0.79	0.14–0.50	0.16–0.54	0.19–0.74	0.17–0.75	0.24–0.94	0.21–0.92
S	22–36	21–31	21–35	21–33	17–26	17–26	11–28	12–27
EPT	1–6	1–6	1–6	1–5	1–7	1–7	1–7	1–7
H′	2.03–2.83	2.11–2.77	1.81–2.45	1.90–2.55	1.76–2.77	1.72–2.83	1.14–2.44	1.28–2.55
Density of invertebrates	280–1384	282–952	285–1232	255–525	280–604	289–571	326–1216	290–453
MMI_PL index	0.547–0.733	0.477–0.700	0.509–0.704	0.501–0.675	0.433–0.780	0.407–0.780	0.392–0.761	0.392–0.771
Ecological quality class	2–3	3	3	3	2–4	2–4	2–4	2–4

Abbreviations: I, spring; II, autumn.

Based on the abiotic classification of Polish surface water types (according to the data sheets for surface waters on the State Water Holding Polish Waters portal), the Nida was identified as a lowland river (type RzN). Over specific years, the ecological status of the Nida River, assessed based on the composition and abundance of benthic macroinvertebrates, was categorized as GES (good ecological status, Class II) and MES (moderate ecological status, Class III) in 2014, MES in 2017, GES and PES (poor ecological status, Class IV) in 2020 and 2023 (Table 3). Statistical analysis revealed significant differences in 1‐GOLD, the total number of families (S), and diversity (H′) values across specific years (ANOVA K‐W; H(3, *N* = 80) = 18.863, *p* = 0.0003 for 1‐GOLD; H(3, *N* = 80) = 38.891, *p* = 0.0000 for S; and H(3, *N* = 80) = 12.802, *p* = 0.0051).

**FIGURE 3:**
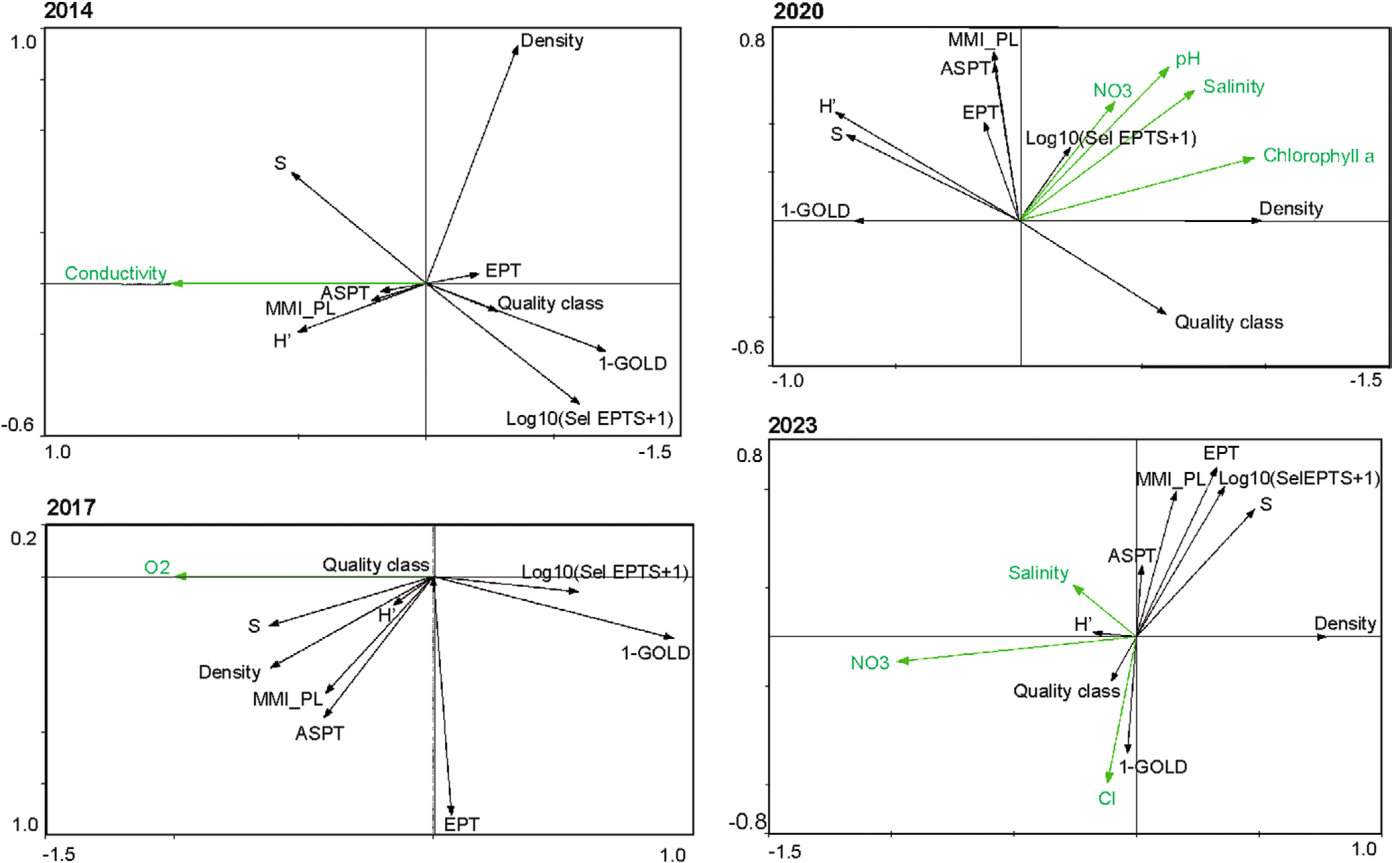
Ordination plots of RDA of component metrics of MMI_PL index and environmental variables during the years. Arrows represent the environmental variables (in green) and MMI metrics (in black).

The findings of our study underscore the critical importance of a long‐term perspective in monitoring studies to effectively utilize benthic macroinvertebrates. Over the years, various parameters impacting the ecological status of river water, index component metrics, and MMI values undergo changes. Additionally, in specific years, the number of variables with a significant relationship to individual component metrics has seen an increase, as demonstrated in the results of the RDA analysis. The analysis unveiled that different environmental variables influenced individual component metrics of the MMI_PL index in successive years (Figure 3). In 2014, conductivity played a significant role, while in 2017, it was dissolved oxygen. By 2020, several crucial factors came to light: pH, nitrate concentration in the water, salinity, and chlorophyll *a*. In 2023, salinity and nitrates notably affected the composition of biological metrics, along with chlorides (Figure 3).

**FIGURE 4:**
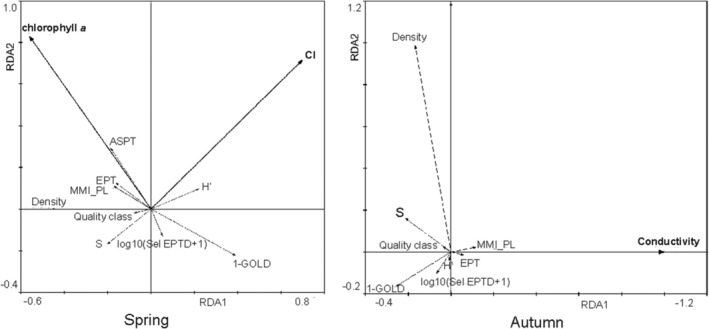
Ordination plots of RDA of component metrics of MMI_PL index and environmental variables in different seasons. Arrows represent the environmental variables and MMI metrics.

Results from RDA in different seasons indicated that chlorophyll a and chlorides (in spring) and conductivity (in autumn) significantly influenced seven component metrics and the water quality class (Figure 4). These results are confirmed by the values of Spearman rank correlation coefficients (spring: chlorophyll a and ASPT *r*_*s*_ = 0.441 and chlorides and 1‐GOLD *r*_*s*_ = −0.403; autumn: conductivity and S *r*_*s*_ = −0.578 and conductivity and H′ *r*_*s*_ = −0.457; *p* < 0.05). In the RDA, including all environmental variables, the first two axes explained from 42.1% to 56.3% of the variability in the data on the composition of biological metrics, and the first axis contributed the majority (from 81.5% to 94.6%), indicating a strong correlation between the environmental variables and biological metric data. Monte Carlo permutation tests showed that the ordination axes of the RDA were significant (Table 4). Discrepancies in the values of component metrics were observed in specific years; however, the water quality class, determined through the use of benthic invertebrates in the two research seasons, remains comparable in the long‐term perspective. The choice of the monitoring season over the years does not significantly impact the value of the MMI_PL index and the quality class; nevertheless, individual component metrics are influenced by various environmental factors.

**TABLE 4:** Results of RDA analyses based on invertebrates, component metrics, and environmental variables.

Parameters	Axis 1	Axis 2	Axis 3	Axis 4	Monte Carlo permutation test of significance of RDA axes
2014					
Eigenvalues	0.319	0.102	0.028	0.306	Axis 1 *F* = 40.6, *p* = 0.001 All axis *F* = 15.8, *p* = 0.001
Species–environment correlations	0.738	0.648	0.696	0.000	
Cumulative percentage variance:					
Of biological data	31.9	42.1	44.9	75.5	
Of biological–environmental relationship	71.0	93.7	96.6	98.7	
2017					
Eigenvalues	0.354	0.209	0.066	0.059	Axis 1 *F* = 42.7, *p* = 0.001 All axis *F* = 26.7, *p* = 0.001
Species–environment correlations	0.832	0.827	0.817	0.938	
Cumulative percentage variance:					
Of biological data	35.4	56.3	62.9	68.8	
Of biological–environmental relationship	51.2	81.5	91.0	99.6	
2020					
Eigenvalues	0.275	0.156	0.021	0.004	Axis 1 *F* = 40.5, *p* = 0.001 All axis *F* = 21.4, *p* = 0.001
Species–environment correlations	0.816	0.590	0.935	0.248	
Cumulative percentage variance:					
Of biological data	27.5	43.1	45.2	45.5	
Of biological–environmental relationship	60.4	94.6	95.2	99.9	
2023					
Eigenvalues	0.352	0.099	0.028	0.270	Axis 1 *F* = 32.6, *p* = 0.001 All axis *F* = 18.4, *p* = 0.001
Species–environment correlations	0.773	0.644	0.683	0.000	
Cumulative percentage variance					
Of biological data	35.2	45.1	48.0	75.0	
Of biological–environmental relationship	73.5	94.1	96.6	98.9	
Spring					
Eigenvalues	0.214	0.061	0.040	0.016	Axis 1 *F* = 39.7, *p* = 0.001 All axis *F* = 19.7, *p* = 0.001
Species–environment correlations	0.750	0.665	0.350	0.461	
Cumulative percentage variance					
Of biological data	21.4	27.5	31.5	33.1	
Of biological–environmental relationship	62.7	80.6	92.3	96.9	
Autumn					
Eigenvalues	0.107	0.094	0.046	0.013	Axis 1 *F* = 34.6, *p* = 0.001 All axis *F* = 10.7, *p* = 0.001
Species–environment correlations	0.694	0.458	0.595	0.500	
Cumulative percentage variance					
Of biological data	10.7	20.0	24.7	26.0	
Of biological–environmental relationship	39.4	74.0	91.1	96.0	

## 4 Discussion

This study addresses a knowledge gap regarding the long‐term approach to ecological status assessment in rivers with agricultural catchments, using benthic invertebrates as indicators. The studies existing to date are mainly technical reports of administrations. Scientific studies and analysis of ecological status using benthic invertebrates have not been provided in the long‐term approach. To date, the utility and convenience of these organisms as ecological indicators have been studied and analyzed, but not in the long‐term context. The eutrophication process driven by high nutrient content is a characteristic feature of numerous European waters. Over 60% of surface waters in Europe fail to achieve the primary water management goal, that of good ecological status, with diffuse emissions from agriculture being the second most significant pressure affecting them (Nikolaidis et al. 2022). Our study affirms the crucial nature of the long‐term perspective in monitoring studies involving benthic macroinvertebrates as parameters impacting the ecological status of river water, index component metrics, and MMI values evolve over time. Our findings are particularly pertinent given the serious threats to the biodiversity and ecological health of river ecosystems (Cardinale et al. 2012; Ruaro et al. 2020; Albert et al. 2021; Laini et al. 2022). In the multimetric approach, metrics are selected and scored, and this study emphasizes their behavior over the long term, crucial in transitioning conditions from natural to degraded (Whittier et al. 2007).

Various indicator metrics and tools are employed in assessing the ecological status of water, including organic pollution assessments using benthic macroinvertebrates (Hettige et al. 2023). Utilizing indicators applicable across national borders is preferable in water quality assessment methods (Von Der Ohe et al. 2007), highlighting the importance of benthic macroinvertebrates as a tool in monitoring studies. They constitute a crucial biotic element of water systems and play a primary role in the ecological assessment of rivers and overall aquatic ecosystem health (Tampo et al. 2021). Changes in their presence serve as indicators of habitat and overall river degradation, reflecting the diversity of anthropogenic disturbances (Von Der Ohe et al. 2007; Fernández et al. 2018). Habitat heterogeneity in streams and rivers can influence the functional composition and diversity of macroinvertebrate assemblages (Sripanya et al. 2023). Given that the serious threats to the biodiversity and ecological health of river ecosystems are seriously threatened (Cardinale et al. 2012), accurately assessing assemblage structure and aquatic ecological health is essential for quantifying the magnitude of degradation and discerning different stressors (Hillebrand et al. 2018).

Benthic invertebrates are considered reliable indicators of long‐term environmental changes due to their persistent presence and limited range. Hence, the long‐term perspective of their use, as demonstrated in our research, proves beneficial for estimating the ecological status of rivers. The MMI index takes into account the effects of multiple anthropogenic impacts on benthos‐fauna metrics, considering biological attributes such as taxa density and richness, composition, pollution tolerance, occupied habitats, and functional feeding. It aggregates individual metrics into a single value for assessing the water quality and health conditions of aquatic ecosystems (Sripanya et al. 2023). Based on the results presented in this study, it can be concluded that the water quality varied in the long‐term perspective. In specific years, the ecological status of the Nida River, determined by the composition and abundance of benthic macroinvertebrates, was categorized as GES (Class II) and MES (Class III) in 2014, MES in 2017, GES and PES (Class II and IV) in 2020 and 2023. Statistical analysis confirmed significant differences in 1‐GOLD, the total number of families (S), and diversity (H′) values across these particular years. A moderate ecological status suggests minor changes in the composition and the number of benthic fauna, indicating moderate alterations in the values of biological elements at the selected sampling points compared to the values established for maximum ecological status. Furthermore, the values of biological elements are more impacted than those determined for good ecological status. Notably, specific taxonomic groups are absent in this type of water, and there is a reduced presence of macroinvertebrate taxa sensitive to disturbances with an increased representation of taxa resistant to disturbances. MMI metrics typically encompass a broad spectrum of biological characteristics and responses to natural gradients and anthropogenic disturbances (Ruaro et al. 2020).

Human activities significantly influence aquatic environments, often surpassing natural processes (Cendrero et al. 2020). In rivers, the escalating pollution is primarily attributed to pollution discharges (Karaouzas et al. 2021; Marrugo‐Negrete et al. 2021), creating a global impact with toxic and lethal consequences for aquatic organisms. The increased pollution levels in agricultural catchments stem from fertilizers, pesticides, and industrial waste discharges from human sources (Li et al. 2019). As was demonstrated previously (Bunn and Davies 2000; James and Marcus 2006), these pollutants have led to the degradation of river functioning, a decline in taxonomic richness, and an overall reduction in diversity, posing a significant threat to river integrity. The Nida River exhibited variations in physico‐chemical water parameters. Our results revealed differences in pH, temperature, salinity, and the concentration of nitrates, ammonia, total dissolved solids, chlorides, dissolved oxygen, and chlorophyll *a* between the study years. The highest value of nitrates was 19.6 mg/dm^3^ and that of ammonia 0.73 mg/dm^3^.

According to the WFD, achieving good ecological status in all waters by 2027 is a goal with less stringent objectives defined only in areas where it is deemed unattainable. Current data from European rivers reveal that only 41% of rivers are in high and good status, 36% in moderate status, and 17% in poor and greatest influence influenced or threatened by various anthropogenic stresses (EEA 2018; Best and Darby 2020). One potential factor impacting the current ecological condition of the Nida River is the occurrence of illegal pollutant discharges. Studies by Lemm et al. (2021) highlight that issues affecting Europe's river waters are quite similar, irrespective of the region or river size. Achieving an improved ecological status is a long‐term objective that necessitates a comprehensive approach to recognizing impacts and implementing management solutions tailored to specific stressors at each site (Lemm et al. 2021). In this context, our long‐term approach utilizing a biological index based on invertebrates holds particular significance as it illustrates the changes in the ecological status of the river system. With river water playing a vital role in agricultural activities, as seen in the case of the studied Nida River, the contemporary society's water footprint and economic water usage emphasize the importance of water resource management for building a sustainable ecological civilization (Tian and Wang 2020). Water resources serve as critical components for sustaining both biological and anthropogenic systems (Khan and Zhao 2019). According to Lorenz et al.'s study (Lorenz et al. 2023), agriculture and urban wastewater act as stressors leading to reduced flow and impacting macroinvertebrates and fish more than macrophytes. Nikolaidis et al. (2022) further demonstrated that agriculture primarily contributes to excessive N and P input in rivers and lakes across Europe.

The evaluation of the ecological status of river waters holds significant importance due to the enforcement of European Union laws by member countries. The Nitrates Directive addressing water protection for agricultural nitrate pollution and the WFD, both EU legal regulations, were implemented in Poland through the Water Law Act of July 20, 2017 (Halecki and Stachura 2020). Following the data of the Central Statistical Office in Poland (GUS 2019, 2020), in the monitoring studies that were carried out from 2014 to 2019, a total of 2206 river sections were assessed for ecological status, and 2087 river sections were monitored for chemical status. Monitoring studies were carried out in 3051 river sections. The Nida River is situated in the Vistula River basin within the Upper Vistula water region. Surface water monitoring results from 2016 to 2021 in the Vistula River basin indicate that 82% of river waters (1410 out of 1719) are in poor condition (aPGW https://www.apgw.gov.pl/). Within this region, 94% of rivers are significantly altered by human activities, putting them at risk of failing to meet environmental goals. Hence, comprehensive actions are crucial for the active protection of all waters, encompassing threat identification, elimination, and continuous water quality monitoring to track changes in the aquatic environment. The 2021 assessment of the ecological status of the middle course of the Nida River based on MMI_PL revealed an improvement compared to 2019, indicating good ecological potential. However, the moderate water potential might be influenced by the river section's proximity to hay meadows, its regulation, or its location near suburban structures. The varying ecological condition of the Nida River in different years suggests persistent risks of failing to achieve environmental goals. Long‐term results indicate that the river's ecological status has been dynamic over the years. Likely factors contributing to this variability are illegal discharges of municipal sewage from households, pollution from agricultural areas, and the use of plant protection products and fertilizers. Unlike industrial pollutants, no nearby industrial plants discharge into the Nida River. Vagheei et al. (2023), examining Spanish rivers, predict rising temperatures, reduced precipitation, and discharge, leading to a downgrading of ecological status in these ecosystems. This underscores the urgency of informed decisions for managing and preserving freshwater resources, emphasizing the need for climate change mitigation and adaptation measures at various scales—local, national, regional, and global. The deteriorating ecological condition of the Nida River over the years highlights the increasing negative impact of human activities on the natural environment.

The pollution of river ecosystems is intricately linked to human disturbances stemming from anthropogenic activities and urbanization (Edegbene et al. 2021). These activities have adverse impacts necessitating increased efforts in water assessment by managers (Clifford and Tariro 2005). Rivers are confronted with various forms of micro and macro pollution (Heß et al. 2023), along with morphological degradation, hydrological alterations, and climate changes (Vörösmarty et al. 2010). The European Union's WFD set the ambitious goal of achieving a “good” chemical and ecological status for all waters by 2027. Therefore, adopting a long‐term approach to assess the ecological status of river systems becomes crucial to meet this requirement.

## 5 Conclusions

The study provides insights into the ecological status of the water in the human‐impacted Nida River, which can be considered a model river reflecting agricultural pollution for the monitoring research of riverine ecosystems in the territory of the central part of Europe. This study showed that the ecological status fluctuated over the years, with classifications ranging from good and moderate in 2014, moderate in 2017, to good and poor in 2020 and 2023. The significance of the long‐term monitoring study lies in the evolving impact of parameters on the river's ecological status, index component metrics, and MMI values. The number of variables exhibiting a significant relationship with individual component metrics has expanded over time; therefore, studies revealed a strong need for monitoring studies from a long‐term perspective. While differences in component metric values were observed in specific years, the water quality class remained comparable over the long term in the two seasons. Individual component metrics were influenced by various environmental factors, highlighting the importance of considering these factors in interpreting the results of benthic macroinvertebrate studies. Studies carried out in the long‐term context enabled us to predict its response to the increasing level of anthropogenic pollution. Taking into account the results of this research, we recommend proper river water management for maintaining the diversity of macroinvertebrates and stabilization of the river ecosystem, especially since the Nida River valley is located within the area of Natura 2000, a network of protected areas covering Europe's most valuable and threatened species and habitats. The most important and decisive threat to achieving good ecological status in rivers located in agricultural areas is the pollution runoff from agricultural fields and households into river waters, as well as ineffective waste management (WIOŚ 2018). The results of this study support the conclusions that the identification of benthic invertebrate communities' distribution and ecological status of the water along the course of the rivers is crucial to observing changes in ecosystems, especially in global climate changes. This study can provide a theoretical foundation for the sustainable regulation and management of rivers with agricultural catchments and serve as a reference for other similar watersheds worldwide.

## Author Contributions

**A. Cieplok:** conceptualization (lead), data curation (equal), investigation (equal), writing – original draft (lead), writing – review and editing (lead). **R. Czerniawski:** investigation (equal). **A. Spyra:** data curation (equal), writing – original draft (equal), writing – review and editing (equal).

## Conflicts of Interest

The authors declare no conflicts of interest.

## Supporting information


Data S1.

## Data Availability

The data generated during this study are included in the article.
