# Supplementary material for: Agricultural Pollution as a Driver for the Ecological Status of Rivers in the Long‐Term Perspective of Nida River Assessment (Central Europe)
Source: Ecol Evol. 2025 Jun 16;15(6):e71541. doi: 10.1002/ece3.71541 (PMC12168479; doi:10.1002/ece3.71541)
Supplement: Supplementary file 1 — Data S1. [file ECE3-15-e71541-s001.docx]

**ONLINE RESOURCES**

**SUPPLEMENTARY MATERIAL**

**Agricultural pollution as a driver for the ecological status of rivers in the long-term perspective of Nida River assessment (Central Europe)**

Cieplok A^1^*, Czerniawski R^2^, Spyra A^1^

^1^Faculty of Natural Sciences, Institute of Biology, Biotechnology and Environmental Protection, University of Silesia, 9 Bankowa Str, 40-007 Katowice, Poland; *Corresponding author

^2^Department of Hydrobiology, Institute of Biology, University of Szczecin, Wąska 13, 71-712 Szczecin, Poland.

E-mail address of the corresponding author: anna.cieplok@us.edu.pl

*corresponding author

e-mail address: anna.cieplok@us.edu.pl

Faculty of Natural Sciences,

Institute of Biology, Biotechnology and Environmental Protection,

University of Silesia,

Bankowa 9, 40-007 Katowice

Poland

**Table 1S.** Characteristics of sampling sites along the course of the Nida River.

| **River course** | **No of sampling point** | **Km of river** | **Predominant land**  **use type *** | **Macrophytes** | **Direct catchment** | **Bottom sediments** | **Type of river transformation** |
| --- | --- | --- | --- | --- | --- | --- | --- |
| Upper | 1 | 43.0 | | 111 compact urban development 0.04% | | --- | | 112 loose urban development 7.35% | | 121 industrial areas  0.39% | | 122 communication areas 0.20% | | 124 airport 0.02% | | 131 open-pit mining sites 0.48% | | 132 heaps, dumps 0.03% | | 133 building site 0.13% | | 141 green areas 0.05% | | 142 sport, recreational areas 0.14% | | **211agricultural, arable lands 44.26%** | | 222 plantations 0.19% | | **231 meadow, pastures 11.85%** | | 242 complex crop, plot systems 2.80% | | 243 agricultural areas with natural vegetation 4.38% | | 311 deciduous forests 3.30% | | **312 coniferous forests 16.23%** | | 313 mixed forests 6.07% | | 324 forests, shrubby vegetation 1.56% | | 411 inland marshes 0.09% | | 412 peat bogs 0.01% | | 512 water bodies 0.44% | | *Potamogeton natans*  *Stuckenia pectinata*  *Lemna minor*  *Galium palustre*  *Glyceria maxima* | meadows | sandy-muddy with high organic matter content | not transformed |
|  | 2 | 53.9 |  | *Phragmites australis*  *Elodea canadensis*  *Myriophyllum verticillatum* | meadows and agricultural areas | sandy with low organic matter content | not transformed |
|  | 3 | 65.5 |  | *Myriophyllum verticillatum*  *Lemna minor*  *Potamogeton crispus*  *Sagittaria sagittifolia*  *Mentha aquatica*  *Phragmites australis*  *Rumex hydrolapathum*  *Lycopus europaeus*  *Lysimachia thyrsiflora* | farm buildings, stud farm, pastures. | sandy with low organic matter content | regulated |
| Middle | 4 | 73.7 |  | *Lemna minor*  *Potamogeton lucens*  *Mentha aquatica*  *Phragmites australis*  *Lysimachia thyrsiflora* | meadows | sandy with low organic matter content | partially regulated |
|  | 5 | 79.2 |  | *Sagittaria sagittifolia*  *Myriophyllum verticillatum*  *Myriophyllum spicatum*  *Potamogeton natans*  *Potamogeton crispus*  *Sparganium erectum*  *Nuphar lutea* | trees and shrubs | sandy with additional rocky and gravel fractions with low organic matter content | partially regulated |
|  | 6 | 85.8 |  | *Phragmites australis*  *Glyceria maxima*  *Sagittaria sagittifolia*  *Mentha aquatica* | urban agglomeration, aeroclub | sandy with low organic matter content | partially regulated |
| Lower | 7 | 101.0 |  | *Potamogeton natans*  *Glyceria maxima*  *Phragmites australis*  *Potamogeton pectinatus*  *Lemna minor*  *Myriophyllum spicatum* | agricultural fields, | sandy with low organic matter content | not transformed |
|  | 8 | 121.6 |  | *Phragmites australis*  *Glyceria maxima*  *Myriophyllum spicatum*  *Sparganium erectum* | agricultural fields, | sandy and muddy  with low organic matter content | partial regulation |
|  | 9 | 131.5 |  | *Phragmites australis*  *Mentha aquatica*  *Carex paniculata*  *Eleocharis palustris*  *Alisma plantago-aquatica*  *Sparganium erectum*  *Potamogeton natans*  *Sagittaria sagittifolia*  *Myriophyllum verticillatum* | agricultural fields, pastures | sandy with low organic matter content | partial regulation |
|  | 10 | 145.9 |  | - | trees and shrubs, road | sandy and muddy, covered with boulders and stones with a high content of organic matter | regulated |

* based on data contained in CORINE Land Cover (CLC 2018)

The predominant share of the catchment area in bold

CORINE Land Cover – CLC. 2018 [https://clc.gios.gov.pl/index.php/o-clc/program-clc data:10.06.2024]

**POLISH MULTI-METRIC INDEX OF ECOLOGICAL STATUS OF RIVERS (MMI_PL)**

*According to: Bis B, Mikulec A, Bielczyńska A (2020) Makrozoobentos w rzekach. Podręcznik do monitoringu elementów biologicznych i klasyfikacji stanu ekologicznego wód powierzchniowych. Aktualizacja metod. Inspekcja Ochrony Środowiska. Biblioteka Monitoringu Środowiska. Warszawa s. 113-159*

The value of the Multimetric Index of the Ecological Status of Rivers (MMI PL) is a weighted average of six partial metrics, which consist of:

1. **ASPT_PL_** is an indicator that is an average score for all families, according to the formula:

$${ASPT}_{PL}=\frac{{BMWP}_{PL}}{n}$$

where:

BMWP_PL_ – is the sum of points assigned to all families identified in the sample at a given sampling point expressed by:

$${BMWP}_{PL}= \sum_{i=1}^{n} B_{i}$$

n – is the number of identified families (assigned a B value)

B_i_ – is the value B assigned to the i-th family

1. **Log_10_(sel_EPTD+1),** which is calculated based on representatives of selected families Ephemeroptera, Plecoptera, Trichoptera and Diptera, according to the formula:

$${Log}_{10}\left( {sel}_{EPTD}+1 \right)={log}_{10}(sum of individuals from Heptageniidae,$$

$$Ephemeridae, Leptophlebiidae, Brachycentridae, Goeridae, Polycentropodidae,$$

$$Limnephilidae, Odontoceridae, Dolichopodidae, Stratiomyidae, Dixidae, Empididae,$$

$$Athericidae, Neumouridae+1$$

2. **1-GOLD,** which is an indicator calculated on the basis of the share of groups of invertebrates resistant to pollution: Gastropoda, Oligochaeta, and Diptera. It is the only indicator among the metrics that decreases with the increase in water quality (GOLD), so it is necessary to use the difference from one to reverse this relationship and use it together with the other metrics:

$$\boldsymbol{1-GOLD=1-}\frac{\boldsymbol{number of specimens from Gastropoda+Oligochaeta+Diptera}}{\boldsymbol{number of specimens in the entire sample(N)}}$$

3. **The total number of families (S)** specified at a given sampling point,
4. **Number of group families (EPT),** which determines the number of families of sensitive groups Ephemeroptera, Plecoptera and Trichoptera at a given measuring station,
5. **The Shannon-Wiener Index (H'),** calculated on the basis of the ratio of the size of a given family to the total number of families present at a given measurement site, takes into account the total number of families, as well as the dominance structure between them

**List of abbreviations**

WDF - Water Framework Directive

NO3 - nitrates

NH4 - ammonia

Cl - chlorides

TDS - total dissolved solids

DO - dissolved oxygen

MMI_PL - Polish Multimetric Index

ICMi - Multimetric Intercalibration Index

RzN - lowland river

ASPT_PL - average score per taxon PL

1-GOLD - abundance of Gastropod, Oligochaeta and Diptera

log10 (sel_EPTD + 1) - selected families from Ephemeroptera, Plecoptera, Trichoptera and Diptera

S - total number of families

EPT - number of families from Ephemeroptera, Plecoptera, Trichoptera

H′ - Shannon-Wiener diversity index

RDA - Redundancy Analysis

GES - good ecological status

MES - moderate ecological status

PES - poor ecological status

**Table 2S.** Ecological Status Classification Method. Limit values ​​for surface water quality classes and surface water quality indicator (macroinvertebrates) for river type RzN.

*According to: Regulation of the Minister of Infrastructure of 25 June 2021 r. (Dz.U. z 2021 r., poz. 1475)*

| Quality class | I | II | III | IV | V |
| --- | --- | --- | --- | --- | --- |
| Benthic macroinvertebrates based MMI_PL | ≥ 0.913 | ≥ 0.710 | ≥ 0.473 | ≥ 0.237 | < 0.237 |
